# Acceptance and Commitment Therapy preceded by an experimental Attention Bias Modification procedure in recurrent depression: study protocol for a randomized controlled trial

**DOI:** 10.1186/s13063-018-2515-9

**Published:** 2018-03-27

**Authors:** Tom Østergaard, Tobias Lundgren, Robert Zettle, Rune Jonassen, Catherine J. Harmer, Tore C. Stiles, Nils Inge Landrø, Vegard Øksendal Haaland

**Affiliations:** 1 Department of Psychiatry, Sørlandet Hospital, Arendal, Norway; 2 Department of Clinical Neuroscience, Center for Psychiatry Research, Karolinska Institute, Stockholm Health Care services, Stockholm, Sweden; 3 Department of Psychology, Wichita State University, Wichita, KS USA; 4 Clinical Neuroscience Research Group Department of Psychology, University of Oslo, Oslo, Norway; 5 Psychopharmacology and Emotional Research Lab (PERL), University Department of Psychiatry, Oxford, UK; 6 Department of Psychology, Norwegian University of Science and Technology (NTNU), Trondheim, Norway

**Keywords:** ACT, ABM, Depression

## Abstract

**Background:**

This project studies the effect of group-based Acceptance and Commitment Therapy (ACT) following Attention Bias Modification (ABM) on residual symptoms in recurrent depression. ACT is a cognitive-behavioral intervention combining acceptance and mindfulness processes with commitment and behavior-change processes. ACT enjoys modest empirical support in treating depression and has also shown promising results in secondary prevention of depression. The experimental cognitive bias modification (ABM) procedure has been shown to reduce surrogate markers of depression vulnerability in patients in remission from depression. The aim of the current project is to investigate if the effect of group-based ACT on reducing residual depressive symptoms can be enhanced by preceding it with ABM. Also, assessment of the relationship between conceptually relevant therapeutic processes and outcome will be investigated.

**Methods/design:**

An invitation to participate in this project was extended to 120 individuals within a larger sample who had just completed a separate randomized, multisite, clinical trial (referred to hereafter as Phase 1) in which they received either ABM (*n* = 60) or a control condition without bias modification (*n* = 60). This larger Phase-1 sample consisted of 220 persons with a history of at least two episodes of major depression who were currently in remission or not fulfilling the criteria of major depression. After its inclusion, Phase-1 participants from the Sørlandet site (*n* = 120) were also recruited for this study in which they received an 8-week group-based ACT intervention. Measures will be taken immediately after Phase 1, 1 month, 2 months, 6 months, and 1 year after the conclusion of Phase 1.

**Discussion:**

This study sequentially combines acceptable, nondrug interventions from neuropsychology and cognitive-behavioral psychology in treating residual symptoms in depression. The results will provide information about the effectiveness of treatment and on mechanisms and processes of change that may be valuable in understanding and further developing ABM and ACT, combined and alone.

**Trial registration:**

ClinicalTrials.gov, Identifier: NCT02648165. Registered on 6 January 2016.

**Electronic supplementary material:**

The online version of this article (10.1186/s13063-018-2515-9) contains supplementary material, which is available to authorized users.

## Background

Depression is a condition that affects many individuals in adverse ways. The World Health Organization Global Burden of Disease Study ranked depression as the single most burdensome disease in the world in terms of total disability-adjusted years among people in the middle years of life [1]. Although many new approaches to treating depression have been developed, efficacy for both pharmacological [2] and psychological interventions [3] is still unsatisfying.

Depression is also a highly recurrent disorder. More than 75% of patients diagnosed with major depressive disorder (MDD) experience more than one episode, often relapsing within 2 years of recovery [4]. National Institute of Health and Clinical Excellence [5] has identified secondary prevention as a key goal in the long-term management of depression. Its high recurrence rate suggests specific vulnerability factors that increase risk for developing repeated episodes of MDD. Preventive strategies that identify and ameliorate these factors ostensibly could reduce risk of subsequent episodes [6].

To achieve a comprehensive understanding of the phenomena of depression and its efficacious treatment, multiple perspectives and angles may be necessary [7]. In a recent article, Beck and Bredemeier [7] proposed a more unified model of depression, integrating clinical, cognitive, biological and evolutionary perspectives for treating and preventing depression. Consistent with this recommendation, this project evaluates the impact in reducing residual symptoms in depression of group-based Acceptance and Commitment Therapy (ACT) following Attention Bias Modification (ABM). ACT has been recognized as an efficacious treatment for depression and has also shown promising results in the secondary prevention of depression (e.g., [8–12]). The experimental cognitive bias modification (ABM) procedure has been shown to reduce surrogate markers of depression vulnerability in patients in remission from depression [13].

The current project is an offshoot of a larger randomized controlled, double-blinded clinical trial (i.e., Phase 1) with an outpatient population with a history of depression (NCT02658682). The original NCT investigated symptom changes after 2 weeks of ABM training. The current trial will investigate the effect on reducing residual symptoms in depression of preceding group- based ACT with ABM.

### Attention Bias Modification

The cognitive model of depression is an empirically based framework for identifying and understanding factors that maintain an episode of depression [14].

Within this model, biased attention is believed to play a key role in maintaining depression by fuelling negative thoughts and feelings. Such effects have been shown using a dot-probe paradigm where participants are presented with pairs of stimuli, typically words or faces, consisting of one neutral stimulus and one emotional stimulus. After the offset of each pair, a dot probe appears in the location of either the neutral or the emotional stimulus with allocation of attention measured by latency in detecting it. Participants orienting selectively toward the emotional stimulus will be faster to detect dot probes that replace that stimulus (where they are already attending) and slower to detect probes that replace the neutral stimulus. For example, it has been demonstrated that clinically depressed subjects orient attention toward sad faces rather than neutral or positive faces [15]. A bias towards sad faces has also been reported in previously depressed, currently euthymic subjects [16], and in never-depressed individuals at high risk because of a family history [17]. In our own research group we have shown similar effects in healthy subjects at genetic risk [18]. Together these results suggest that negative cognitive biases may constitute important vulnerability factors for depression, rather than simple markers of lowered mood.

#### The Attentional Bias Modification procedure

**Fig. 1:**
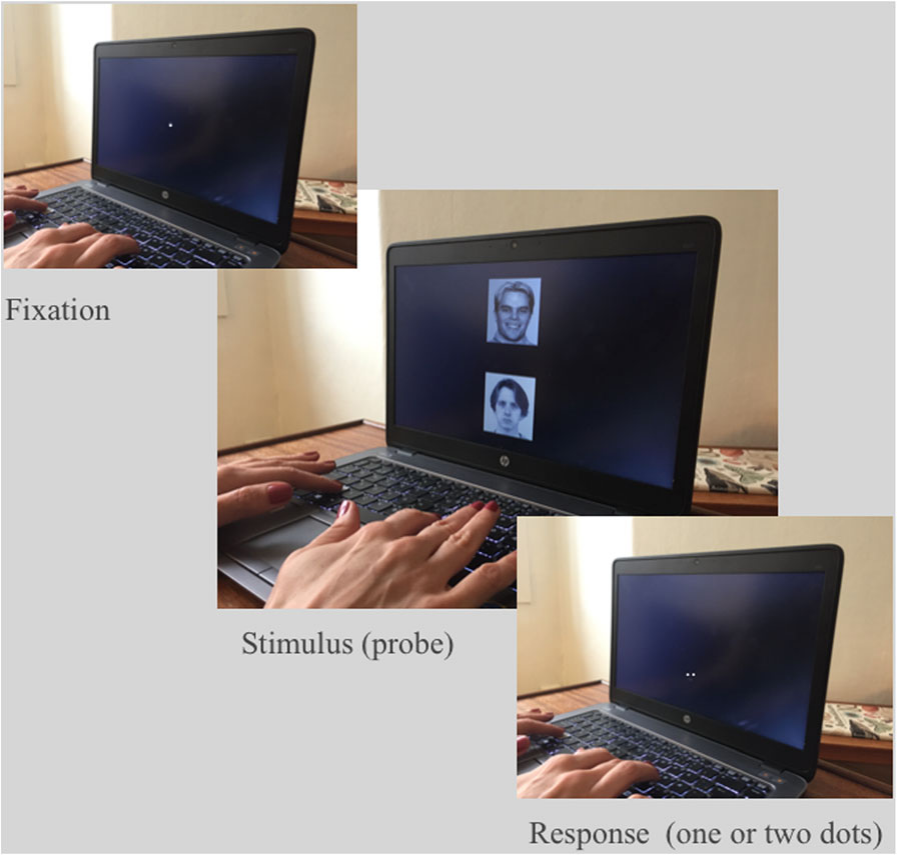
Illustration of the dot-probe paradigm. Attentional bias is reflected in faster times to respond to probes presented in the same position as the negative facial expression versus probes located in the opposite position

Selective biases in attention can be modified by a simple computerized technique; the Attention Bias Modification task (ABM), pioneered by Colin MacLeod et al. [19], was originally developed to test the critical hypothesis that attention bias towards negative emotional information plays a causal role in depression and anxiety [19]. The current approach uses a variant of the dot-probe task, which encourages patients to orient attention towards positive rather than negative stimuli, through an implicit association between the valence of the stimulus and the location of the probe. If the probe appears in the location of the positive stimulus (in 80% of the trials), a habit of automatically directing attention toward positive stimuli is encouraged; i.e., the subject develops a positive attention bias. This manipulation can be compared to a neutral (“placebo”) condition in which the probe appears equally often behind the positive and negative stimuli, without affecting attention bias Fig. 1.

There has been a growing interest in ABM research [20]. Unfortunately, some projects have been limited by small sample sizes and poor trial methodology [20–23]. While overall findings have been mixed, studies that have found measurable positive changes in emotional biases with ABM, have also reported associated changes in clinical symptoms [13, 24, 25]. For example, a recent small-scale study from the research group of Harmer revealed that alteration of attention bias using the ABM technique causally influenced residual symptoms and the cortisol awakening response [13].

### Acceptance and Commitment Therapy

Acceptance and Commitment Therapy (ACT) is a modern cognitive-behavior therapy that combines acceptance and mindfulness processes, with commitment and behavior-change processes. ACT is a transdiagnostic model that focuses on pathogenic processes that are hypothesized to be common in different forms of human suffering [26]. The main goal in ACT is to foster psychological flexibility, which is defined as “the ability to contact the present moment more fully as a conscious human being and to change, or persist in, behavior when doing so serves valued ends” ([27], p. 7). Psychological flexibility has shown to be associated with higher life satisfaction, emotional well-being, job performance, and job satisfaction [28–31]. Because ACT focuses on promoting psychological flexibility, rather than removing psychopathology, ACT is hypothesized to be well suited for the prevention of psychological disorders [32]. Psychological flexibility, in turn, is strengthened through the six core processes of (1) acceptance, (2) defusion, (3) self as context, (4) committed action, (5) values, and (6) contact with the present moment [32]. Of these six process, acceptance has received the most attention. Correlational and experimental evidence indicates that experiential avoidance, as the converse of acceptance, contributes to the development and maintenance of different psychological and behavioral problems [32]. A basic tenet of ACT is that by nurturing and developing a more accepting and psychologically flexible stance makes it possible to remain in contact with the uncomfortable experiences, while remaining focused on what one wants life to be about both now, and in the future. Another process contributing to psychological flexibility that ACT addresses, that would appear to be of greater relevance for the rationale of this study, is that of present-moment awareness. To somewhat varying degrees and via differing means, ACT protocols seek to strengthen mindfulness in a manner consistent with that of Jon Kabat-Zinn [33] as “paying attention in a particular way: on purpose, in the present moment, nonjudgmentally” (p. 4). Depressed clients are encouraged to “just notice” with an accepting attitude thoughts, emotions, and bodily sensations that might otherwise be responded to in a ruminative and experientially avoidant manner.

#### The ACT model of depression

From an ACT point of view, depression is a secondary emotion that emerges from unsuccessful efforts to experientially control sadness and disappointment as normal and adaptive emotional reactions to distressing life events [26]. In effect, sadness that is not toxic or psychologically unhealthy per se, may be transformed into clinical depression. Rather than targeting changes in the thinking of those who struggle with depression, ACT seeks to change how they respond or relate to their thoughts. For example, thoughts like “I don’t deserve to be loved” may often lead to social isolation. Instead of changing the thought, ACT through mindfulness, acceptance, and defusion strategies seeks to minimize its impact as a barrier to psychological flexibility. In depression there is often a reduction in pleasurable and task-oriented activities [14]. From an ACT perspective, it is not an increase in activity levels by itself that is most important. It is rather exploring and clarifying values, and exemplifying psychological flexibility by committed actions coherent with values in multiple life areas [9]. Consistent with this formulation, Plumb, Hayes, Hildebrandt and Martin [34] found an association between depression and lack of valued action.

### Combining ACT and ABM to reduce the likelihood of recurrence of depression

The overarching hypothesis of this project is that because both ABM and ACT seek to increase attentional flexibility, albeit in differing ways and perhaps to differing degrees, they are likely to complement each other. Accordingly, participants who receive sequentially both treatments generally are expected to demonstrate greater benefits than those who only receive ACT. In a special issue on ABM, Koster and Bernstein suggests that combining ABM and clinical treatment might give a better outcome than “stand-alone” treatment [35].

A positive attentional bias established and maintained through ABM should reduce the degree to which negatively valenced stimuli (whether they be sad faces or unwanted thoughts and feelings) become the focus of present-moment awareness. If successful, ABM, in effect, should thus result in less attentional material that is subject to rumination. However, ABM alone does not address how individuals may continue to process and react to negatively valenced material that is attended to in a judgmental way. To the extent that ACT complements ABM, it may primarily do so through its emphasis on mindfulness. In short, individuals who receive both ABM and ACT may be less likely to (1) even be aware of stimuli and events that might otherwise trigger rumination and (2) to be more accepting of that which is still noticed. Studies have found that both ABM and mindfulness-based treatments (of which ACT is an example) may cause neuroplastic changes in the brain regions associated with attention, emotion and self-awareness [24, 36–39]. The changes that ABM might bring forward could predict a boost to ACT treatment.

Another lens through which ABM and ACT could be seen as complementing each other entails multilevels of cognitive processing. ABM could be thought to involve “lower-order” cognitive processes incorporating implicit attention without the involvement of apparent language or cultural-based processes. ACT, on the other hand, implicates more “higher-order” cognitive processes. Somewhat relatedly, Skinner’s distinction between contingency-shaped (ABM) and rule-governed behavior (ACT) [40] may also be useful in considering how ABM and ACT could work together. ABM targets contingency-shaped behavior. In the dot-probe paradigm attention under the discriminative control of positive stimuli is shaped to establish a positive bias. Once established at sufficient strength, selective attending may be maintained by its naturally reinforcing consequences. In contrast to ABM, ACT is more concerned with rule-governed behavior. Mindfulness instruction and practices in ACT can be viewed as efforts to increase attentional flexibility by bringing certain facets of present moment awareness under more deliberate control. However, unlike in ABM, the agenda is less on shifting from *what* is attended to, to that of altering *how* whatever is noticed is attended to in a less judgmental and more accepting manner.

### Hypotheses

This study has the following hypothesis;

1. Group-based ACT will reduce residual symptoms of depression when compared to patients not receiving group-based ACT
2. Group-based ACT will reduce cognitive, neurobiological, and emotional markers of vulnerability in a sample of patients in remission from depression when compared to patients not receiving group-based ACT
3. Group-based ACT preceded by ABM will have greater effect on reduction of residual symptoms than group-based ACT proceeded by sham ABM
4. The absolute and relative reductions in episodes of low-mood among those receiving group-based ACT will be maintained over a 12-month follow-up period
5. Increased valued living and mindfulness skills as well as decreased scores on perceived stress, experiential avoidance, automatic thoughts and cognitive fusion, alone or in combination, will significantly mediate reductions in depressive symptoms in group-based ACT

## Methods/design

### Overview of study design

**Fig. 2:**
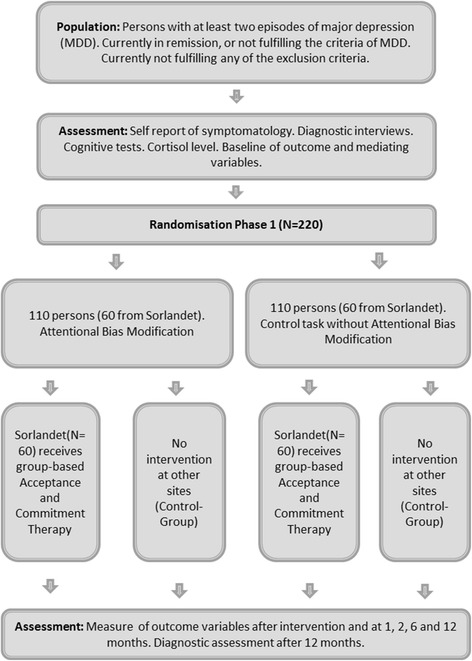
Overview of study design

Figure 2 illustrates the design of the study. Former depressed participants and those in remission (*n* = 220) will initially be randomized in Phase 1 to receive either ABM or a control condition without bias modification. All participants recruited at Sørlandet (*n* = 120), half of whom will have just received ABM, will next receive an 8-week, group-based ACT intervention. The dependent variables are residual symptoms of depression, cognitive, neurobiological, and emotional markers of vulnerability, as well as frequency of major depressive episodes over a 12-month follow-up. The SPIRIT (Standard Protocol Items: Recommendations for Interventional Trials) for this research is available in Additional file 1.

### Participants

Participants with a history of major depression will be recruited by referrals. While referrals are accepted from any source, the majority are expected to come from local hospitals, regular general practitioners, and self-referrals. The age of the participants will range from 18 to 65 years. Diagnostic assessment and evaluation of remission will be made in accordance with the structured clinical interviews for *Diagnostic and Statistical Manual of Mental Disorders, 4th edition* (*DSM-IV*) criteria (The Mini International Neuropsychiatric Interview (MINI)) [41].

#### Inclusion criteria

Inclusion criteria are as follows:

1. Participants with a history of major depression, currently in remission
2. Aged between 18 and 65 years

#### Exclusion criteria

1. Current or past neurological illness
2. Bipolar disorder
3. Psychosis
4. Drug addiction
5. Attention deficit disorder with and without hyperactivity (ADHD and ADD)

### Sample size and power

An earlier preliminary study using the same ABM procedure [13] found a relatively high effect on the measure of residual symptoms (Hedges’ *g* = 1.32). Based on former group-based ACT interventions, we expect an effect above *d* = 0.50 on measures of residual symptoms [42]. We have no relevant studies that can guide our expectations when it comes to the sequential combination of ABM and ACT compared to ACT alone. However, we hypothesize that the combination should result in a larger effect than the individual interventions. Statistical power analysis using G*Power [43] indicates that with an α-level at 0.05 and, a β-level at 0.10, the total sample size in a design like this should be 206 to detect differences in the main and interaction effects of a moderate to medium effect size, *f* = 0.25, with a one-two-way analysis of variance (ANOVA). We plan to recruit 220 participants in total, which allows for some attrition before the power will be reduced.

### Procedures

#### Randomizing and treatment allocation

**Fig. 3:**
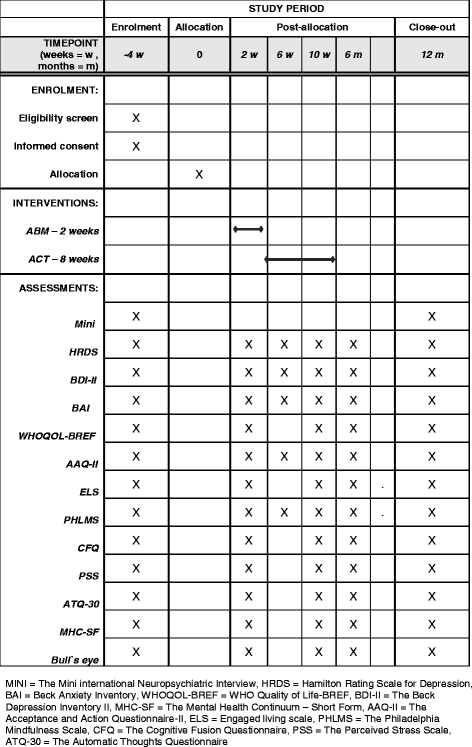
Spirit diagram

**Fig. 4:**
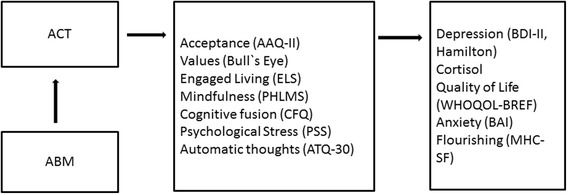
Overview of measures

Following enrollment in Phase 1 and randomization into experimental versus control conditions, tasks (ABM or control) will be completed twice daily over the course of 14 days; i.e., 28 sessions total (see Browning et al., [13] and Fig. 4). Allocation to treatment condition will be done using random-number-generator function in Microsoft Excel by individuals not involved in the recruitment, assessment, treatment, or follow-up of patients. Figure 3 shows the Standard Protocol Items: Recommendations for Interventional Trials (SPIRIT) Figure for the trial process.

Data will be collected in six assessment sessions: (1) immediately before starting Phase 1, (2) immediately after completion of Phase 1, and then at (3) 1 month, (4) 2 months, (5) 6 months, and finally (6) 12 months following Phase 1. The effect of Phase 1 on attentional bias (measured with the dot probe) as a manipulation check will be assessed on the second visit. Participants after Phase 1 (*n* = 120) will receive an 8-week-long ACT-based group intervention. Half of these participants (*n* = 60) will have received ABM during the task phase, while the remainder will be from the control condition Fig. 3.

#### Assessments and measurements

##### Primary outcome

Primary outcome is change in residual symptoms of depression measured at all six assessment sessions using both self- and clinician-rating scales Fig. 4.

The Beck Depression Inventory II (BDI-II) [44] consists of 21 items, and is a strong measure of depressive severity. The Norwegian version of the BDI-II displays high internal consistency, and acceptable convergent and discriminative validity [45].

The Hamilton Rating Scale for Depression (HRDS) [46, 47] is a widely used semi-structured clinical interview measuring the severity of depressive symptomatology covering a range of affective, behavioral and biological symptoms. The HDRS-17 has acceptable psychometric properties [48]. The clinical interview has been conducted blindly and reliably.

##### Secondary outcomes

Secondary outcomes are as following:

- Recurrence of major depressive episodes
  Measured by the MINI structured interview [41] 12 months after baseline
- Changes in symptoms of anxiety
  Change in symptoms of anxiety as measured by the 21-item Beck Anxiety Inventory (BAI) [49]. The BAI has been found to have high internal consistency and has shown good convergent and divergent validity [50, 51]
- Changes in quality of life
  Quality in life is measured by the WHO Quality of Life-BREF (WHOQOL-BREF) [52, 53], which is a 26-item version of the WHOQOL-100 assessment. With satisfactory psychometric properties [52, 53]
- Flourishing
  There is increasing interest in investigating what promotes positive mental health and well-being as a continuum, separate from, albeit related to, mental illness. Flourishing is a term describing subjective well-being [54] which has been operationalized as encompassing facets of (1) emotional, (2) psychological, and (3) social well-being ([55], p. 99). The study uses The Mental Health Continuum – Short Form (MHC-SF) [55], which has been found to have good psychometric properties [55], to measure these three main dimensions of well-being
- Early morning cortisol response
  It is well established that dysfunction of the hypothalamic-pituitary-adrenal (HPA) axis with elevated plasma cortisol levels is characteristic of MDD [56]. Increased secretion of cortisol has also been reported to be present in depressed subjects after clinical recovery [57], and has been suggested to be a vulnerability marker. This study will collect early morning cortisol response, as a natural neuroendocrine challenge test to assess the hypothesis that reduced HPA activity will be a surrogate marker of residual symptoms and thus relapse prevention following ABM and ACT.

##### Mediator measures

The following measures assess potential mediators of change in the interventions. These are psychological variables that are specifically targeted by ACT and that are thought to promote psychological flexibility and mental health functioning:

- Acceptance
  Acceptance is measured by the Acceptance and Action Questionnaire-II (AAQ-II) [58]. AAQ-II has been found to have acceptable structure, reliability, and validity [58]
- Values
  Value-congruent behavior is measured by using Bull’s Eye [59]. Bull’s Eye is a process and outcome measure for treatments that include value-based components. It has shown good temporal stability and has many properties supporting its construct validity [59]
- Engaged living
  Engaged living is a term that is used to describe ways in which valued life activities are pursued [60]. Engaged Living Scale (ELS) [61] is a newly developed measure that addresses the processes of values and committed action from the framework of ACT. A study by Trompetter et al. [61] found ELS to be a valid and reliable measure of an engaged response style
- Mindfulness
  Present-moment awareness and acceptance are two key components of mindfulness. These two key constituents will be measured by the Philadelphia Mindfulness Scale (PHLMS) [62]. The psychometric evidence suggests that PHLMS is a good measure for present-moment awareness and acceptance [62]
- Cognitive fusion
  Cognitive fusion is central process in the ACT model. Cognitive fusion is measured by the Cognitive Fusion Questionnaire (CFQ) [63] which has good psychometric properties based on preliminary findings [63]
- Psychological stress
  Psychological stress is measured by The Perceived Stress Scale (PSS) [64] which has been found to have acceptable psychometric properties [65]
- Automatic thoughts
  Frequency of negative automatic thoughts is measured with the Automatic Thoughts Questionnaire (ATQ-30) [66]. The Norwegian version of the ATQ has been found to have adequate reliability and validity properties [67]
- Other cognitive measures
  Because ABM involves some general cognitive control functions, a measure of basic inhibitory control and mental flexibility will be included; i.e., the Stroop Color Naming Task (D-KEFS; [68]) Fig. 4

##### Moderators

This study will look for possible moderators of therapeutic improvements and relapse; the analyses will, however, be exploratory. These include: level of depression, number of previous depressive episodes, comorbidity, level of education and medication.

#### Data analysis

The primary analyses will follow an intention-to-treat approach using mixed models [69]. To investigate potential confounding variables, comparison conditions will be evaluated for any pre-treatment differences in variables (e.g., gender) and levels of depression using a *t* test. Any such differences will subsequently be treated as covariates (e.g., level of depression) or be included as variables (e.g., gender) within the primary analyses.

To accommodate missing data, mixed models, which involve randomly deleting missing observations without dropping participants, will be used where appropriate in conducting primary analyses [70]. Multiple regressions will be considered to explore potential moderators, and how acceptance, values, engaged living, mindfulness, cognitive fusion, psychological stress and automatic thoughts mediate outcome [71].

#### Therapists and treatment adherence

The ACT group treatment is delivered by clinical psychologists who are trained and have experience in ACT. Therapists will be supervised by experienced ACT therapists, Professor Robert Zettle and PhD Tobias Lundgren. The therapists will meet regularly to review and reflect on treatment procedures and group processes. A manual for ACT group treatment in Norwegian has been developed to enhance treatment compliance and is available upon request. Treatment adherence is investigated by independent ACT researchers. Sessions are video-recorded with the consent of all the participants and knowledgeable ACT researchers that are not involved the treatment groups will check for adherence by randomly reviewing parts of sessions.

#### Interventions

##### ABM task

**Fig. 5:**
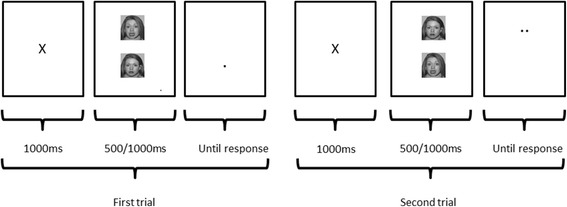
Two example trials from the Attentional Bias Modification task (from Browning et al., [11]). In the experimental group, the probe appears under the most positive stimulus (out of the two examples) on 80% of trials, therefore encouraging increased attention to the positive cues

The ABM task from Browning et al. [13] will be employed using facial stimuli of positive, neutral or negative valence. Each trial will display stimuli from two valences; i.e., three possible stimuli pair types: (1) positive-neutral, (2) positive-negative, and (3) negative-neutral (for details, see [13]) Fig. 5. The patients participate in the training regime twice a day for 2 weeks at home using a laptop provided by us. Prior to the training, patients are shown the program by the researcher and given a practice session on loading and running the task which is set up to allow user-friendly access. At the end of the 2-week period, laptops are collected and data on the compliance with training is collected.

In the control group, there is no contingency between the facial expressions shown and the probe location. However, in the ABM group, the probe appears under the location of the most positive stimulus of each pair (in 80% of trials), thereby encouraging a positive attentional bias. The introduction of this relatively general rule (i.e., most positive of the two stimuli rather based on precise stimuli features), aims to increase generalization of the attentional bias and thereby its longer-term effects. Consistent with this, Browning et al. [13] reported robust effects of ABM on surrogate markers of depression vulnerability beyond narrowly defined measures of attentional bias.

##### ACT group intervention

ACT as a group intervention for secondary prevention of depression is based on the six processes that support psychological flexibility, and is aimed at clients who are in remission from depression. The intervention seeks to increase psychological flexibility and mindfulness, which ACT identifies as essential in developing an engaging and meaningful life. An important treatment target is experiential avoidance, which is thought to “function as a core psychological diathesis underlying the development and maintenance of several forms of psychopathology” ([72], p. 369). The intervention aims at increasing experiential acceptance, which is a posture that reflects openness to both aversive and pleasant experiences, as an alternative to experiential avoidance. Mindfulness exercises are used in every meeting as an exercise to become present and focused in observing what takes place in the present. Each meeting is ended by reviewing what participants are encouraged to practice or reflect on until the next session. Emphasis is placed not on how well they do the exercises that are important, or if they do them at all for that matter, but on their experiences and their observations. Each group of 8–12 participants will receive eight weekly sessions of ACT:

- First meeting
  The first meeting provides an introduction to the central components of ACT and seeks to create an atmosphere of safety, trust, openness and willingness for participants. The objective is to ready for the journey we as a group are to undertake together. This journey involves inviting and making room for difficult emotions and thoughts, in order to focus on what participants want their lives to be about. An engaging and flexible contact with emotions and thoughts has the potential of providing valuable information about oneself, and, furthermore, of releasing energy that previously would be used to try to contain these difficult emotions and feelings. For most of the participants, this perspective represents a shift of paradigms
- Second meeting
  The second meeting has acceptance as a headline, and participants are early on invited into a process of reflection. Acceptance is introduced as an alternative to controlling and avoiding unwanted thoughts, feelings, and sensations. Metaphors and exercises are used to create several experiential examples of acceptance. To make acceptance personally relevant participants are challenged to share personal examples with the group that underscores the workability of acceptance versus avoidance
- Third meeting
  The third meeting has values as a headline. Participants are invited to reflect on what is, and what could be, important and meaningful in their lives. Some perspectives on values are introduced, but not in the sense that we as group leaders have the power of definition. Rather, participants are invited to share their own viewpoints to co-create what values mean, and to make them personally relevant. The connection between acceptance and values is made more explicit and exemplified. Values are often nonverbal (implicit), and this meeting seeks to start the process of identifying values and making them more explicit
- Fourth meeting
  The fourth meeting has defusion as a headline. Defusion is a process of being able to create some distance between ourselves and unwanted psychological experiences in the service of doing what is important, valuable, and meaningful. The process is highlighted by reflecting on how stories about ourselves affect us. The participants engage in exercises designed to increase awareness of the kind of life stories they construct and choose to represent themselves. The life story is then challenged and deconstructed
- Fifth meeting
  Headlines for the day are actions and engagement. Participants are introduced to the notion that people’s lives can be usefully divided into four different central areas or domains: (1) relationships, (2) education and work, (3) health and personal development, and (4) spare time. The remaining meetings will examine participant goals and aspirations in a different life area each time. In this meeting, participants are challenged to select a focus important to them within the broader domain of relationships (e.g., being a loving spouse), and choose congruent short- and long-term goals. Participants are encouraged to set small goals and plan concrete small actions that would bring them a step closer to their values
- Sixth meeting
  The theme for this day is the observing self, which describes a transcending perspective that emerges from noticing our on-going stream of both internal and external experiences. It can be thought of as an invariant vantage point from which we can notice that we notice and see that we see. The observing self explicitly builds around the capacity to see ourselves having thoughts and feelings while not being defined by them. The work with value-based actions continues in the domain of health and personal development
- Seventh meeting
  Headline for the seventh day is self-compassion, which is the capacity to direct kindness and warmth towards yourself and your inner experiences. In this meeting value-based actions are explored within the area of work and education
- Eight meeting
  The eighth session features summarizing and strengthening constructive processes that have been initiated and activated in earlier meetings. Participants are also challenged to investigate and choose value-based actions in the life domain of spare time. An important theme is how to continue the work of developing, exploring, and internalizing the processes that have been most helpful for them

## Discussion

Depression is a highly recurrent disorder, and pharmacotherapy is largely used as an aid to prevent relapse. However, many patients do not wish to use long-term drug treatment and psychological interventions are often a preferred option. Recent evidence suggests that relatively automatic training using ABM can prevent some markers of relapse in formerly depressed patients. ACT has shown promising results in secondary prevention for other patient groups, and is recognized as an empirically supported treatment for depression.

The sequential presentation of these two treatments can be viewed as somewhat controversial as they come from quite different scientific and philosophical traditions. ACT originates from cognitive-behavioral psychology, while ABM originates from neuroscience. Both treatments have had promising effect on depression separately, but their combination has not been investigated before.

While psychological interventions may often be offered in conjunction with antidepressant medication, they are seldom combined with each other. If psychological approaches are combined they are often closely interrelated theoretically and philosophically; unlike ACT and ABM. Paradoxically this may also be why ACT and ABM may work well together. The two treatments both can be seen as targeting attentional processes, but on different levels cognitively, theoretically, and philosophically.

This project takes place in a “real-world” clinical setting, where patients have varied history, backgrounds and challenges. ABM and group-based ACT represent two cost-effective and easily accessible treatments. If combining these two treatments should prove effective, it could represent an interesting opportunity for patients with recurrent depression, and a shift in perception of combining two quite different treatments. To our knowledge there has been one other study where the effect of combining ABM with regular treatment was investigated. Salemink et al. conducted a small randomized study, investigating the combination of cognitive-behavioral therapy (CBT) with ABM, finding that ABM-augmented CBT treatment in adolescents with obsessive-compulsive disorder (OCD) [73]. Additional and larger studies are needed to investigate whether these findings can be replicated. The current ABM and ACT project will hopefully contribute to increasing the understanding of mechanisms in ABM and ACT, and the clinical impact of combining the two.

## Trial status

Recruitment started in April 2015 and is expected to be completed in December 2017.

## Additional file

Additional file 1: ABM/ACT SPIRIT 2013 checklist. (DOC 114 kb)

## Abbreviations

AAQ-II: The Acceptance and Action Questionnaire-II
ABM: Attention Bias Modification
ACT: Acceptance and Commitment Therapy
ADD: Attention deficit disorder
ADHD: Attention deficit hyperactivity disorder
ATQ-30: The Automatic Thoughts Questionnaire
BAI: Beck Anxiety Inventory
BDI-II: The Beck Depression Inventory II
CBT: Cognitive behavioral therapy
CFQ: The Cognitive Fusion Questionnaire
ELS: Engaged Living Scale
HPA: Hypothalamic-pituitary-adrenal
HRDS: Hamilton Rating Scale for Depression
MDD: Major depression
MHC-SF: The Mental Health Continuum – Short Form
MINI: The Mini International Neuropsychiatric Interview
OCD: Obsessive-compulsive disorder
PHLMS: The Philadelphia Mindfulness Scale
PSS: The Perceived Stress Scale
WHOQOL-BREF: WHO Quality of Life-BREF
